# Cardiac myxoma presenting with multisystem involvement

**DOI:** 10.15537/smj.2022.43.9.20220346

**Published:** 2022-09

**Authors:** Ghofran A. Ageely, Salhah S. Alsulami, Ahad A. Alkenani, Ebtihal EMS. Albeshri

**Affiliations:** *From the Department of Internal Medicine, Radiology Division, Rabigh Medical College, King Abdulaziz University, Jeddah, Kingdom of Saudi Arabia.*

**Keywords:** cardiac myxoma, intracranial haemorrhage, carney complex, pituitary apoplexy, adrenal mass

## Abstract

A cardiac myxoma is a rare tumor that could be incidental or present with common symptoms due to embolization. A minority of cases are attributed to carney complex, a rare inherited disease. A 73-year-old Asian male presented with acute left-side weakness, slurred speech, gait imbalance, and subacute constitutional symptoms. Left atrial myxoma was discovered by computed tomography and confirmed by echocardiography. Brain imaging revealed pituitary macroadenoma with subarachnoid and intraventricular hemorrages. The hormonal profile confirmed pituitary apoplexy, for which hormone replacement was initiated. Workup also revealed multiple endocrine tumors and excluded infection and malignancy. Myxoma resection could not be carried out, due to the patient’s rapid clinical deterioration and death.

Furthermore, the presence of cardiac myxoma, non-functioning pituitary macroadenomas, and pituitary apoplexy is extremely rare and rarely documented in the literature. Therefore, we emphasize clinical awareness of rare conditions with atypical presentations to improve outcomes.

Primary tumors of the heart, in general, are considered rare, with an annual incidence of around 0.5 cases per million.^1^ Nearly 75% of cardiac tumors are benign; myxoma is the most common type which accounts for 50%, followed by rhabdomyoma which accounts for 20% of cardiac lesions.^2^ Cardiac myxoma primarily affects women, with patients ranging in age from 4 months to 79 years (mean age: 45 years).^3^ Because myxoma is more common on the left side (75% are in the left atrium), more than half of the embolic events affect the central nervous system and the retinal arteries.^4^ Clinical manifestations of myxoma include cardiac symptoms, constitutional symptoms, and systemic embolic events. As well as, cerebrovascular events including transient ischemic attack, retinal ischemia, and, to a lesser extent, intracerebral hemorrhage, are common first presentations in patients with myxoma.^5^ Even in the absence of a cerebral aneurysm, a patient with cardiac myxoma can experience subarachnoid hemorrhage.^6^ Carney complex (CNC) is a rare autosomal dominant disorder that accounts for less than 10% of cardiac myxoma patients. It is characterized by myxomas, skin pigmentation, and endocrine hyperactivity.^7^ We present a difficult case of atrial myxoma presenting with acute neurological symptoms, pituitary macroadenoma, and pituitary apoplexy.

## Case Report

A 73-year-old Asian male presented to the Emergency Department complaining of left-side weakness that lasted one day. He has hypertension and is taking amlodipine, and he has a history of migraines for which he is taking analgesia.

### Clinical findings

Three weeks before his symptoms began, he had experienced dizziness while driving his car, as well as progressive slurred speech, behavioral changes, and gait imbalance. He has no siblings and unremarkable family history. He was initially admitted as a case of encephalitis. He was afebrile upon admission, with a heart rate of 86 beats per minute, a respiration rate of 20 breaths per minute, and an arterial blood pressure of 120/78 mmHg. His Glasgow coma scale score was 13 (eye-opening response: 3; motor response: 5; and verbal response: 5). A neurological examination revealed bilateral spastic catch and hyperreflexia in the upper and lower limbs, with a normal plantar response. Cerebellar dysdiadochokinesia and dysmetria were also present. Skin examination did not reveal any pigmentations. The sequence of neurological events and related imaging are outlined in Table 1.

**Table 1:** - Sequence of events.

Days	Events
Day one	CT brain showed sellar/suprasellar mass with mild ventricular dilation.
Day 2	MRI brain showed a sellar mass with suprasellar extension and old subarachnoid and intraventricular haemorrhage.
Day 8	CT chest and abdomen and echocardiography revealed multi-organ lesions.
Day 9	The patient developed generalized seizures controlled by Levetiracetam.
Day 10	CT brain revealed acute intraventricular haemorrhage with worsening communicating hydrocephalus.
Day 13	An external ventricular drain was inserted for decompression of the hydrocephalus.
Day 16	He was intubated on mechanical ventilation due to a sudden drop in GCS and worsening seizures. Repeated CT brain showed a new acute parietal intracerebral haemorrhage and MRI revealed no aneurysm or vascular abnormality.
Day 53	Brain MRI showed right occipital acute infarction.
Day 65	CT brain showed a right occipital hemorrhagic transformation.
Day 86	Cardiac arrest and death.

CT: computed tomography, MRI: magnetic resonance imaging, GCS: Glasgow coma scale

### Diagnostic assessment

**Figure 1:**
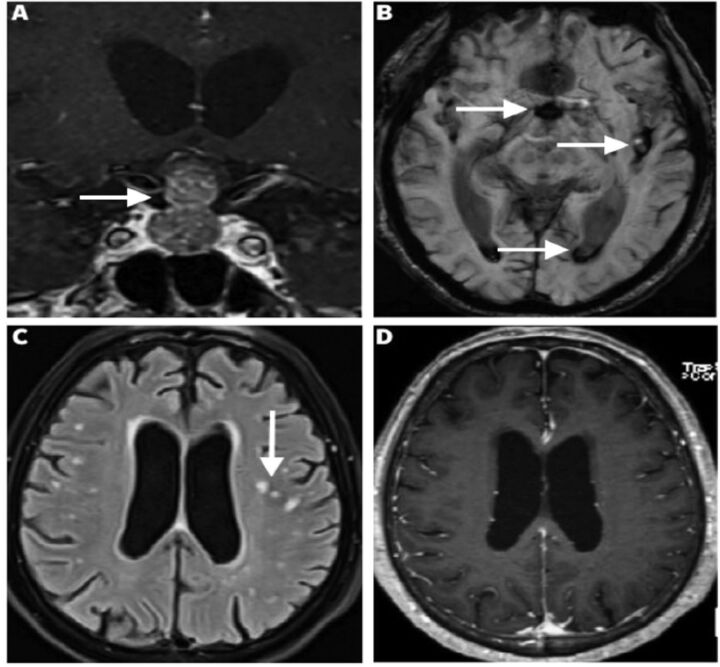
- Brain magnetic resonance imaging showing: A) postcontrast coronal view demonstrating enhancing sellar mass with suprasellar extension giving the Snowman sign, typical for pituitary macroadenoma, there is also mild ventricular dilatation; B) susceptibility weighted imaging revealing blooming in the Sylvian fissure and sulci representing subarachnoid haemorrhage, in the posterior horns of the lateral ventricles representing intraventricular haemorrhage and in the suprasellar part of the pituitary macroadenoma in keeping with pituitary apoplexy; C) FLAIR; and D) postcontrast images demonstrating multiple white matter hyperintense foci without enhancement representing small vessel disease/infarctions.

**Figure 2:**
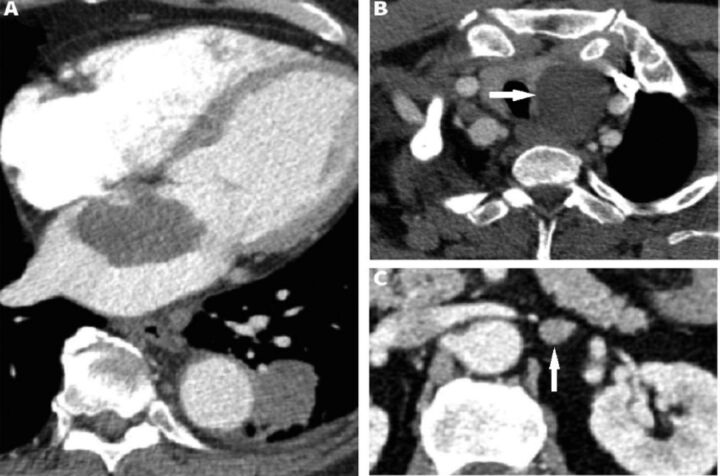
- Enhanced computed tomography axial images demonstrating: A) 4.7 cm left atrial mass based on the interatrial septum at fossa ovalis typical for atrial myxoma, additionally, there is a left lower lobe mass; B) a large left thyroid lobe mass ; and C) a small left adrenal mass.

**Figure 3:**
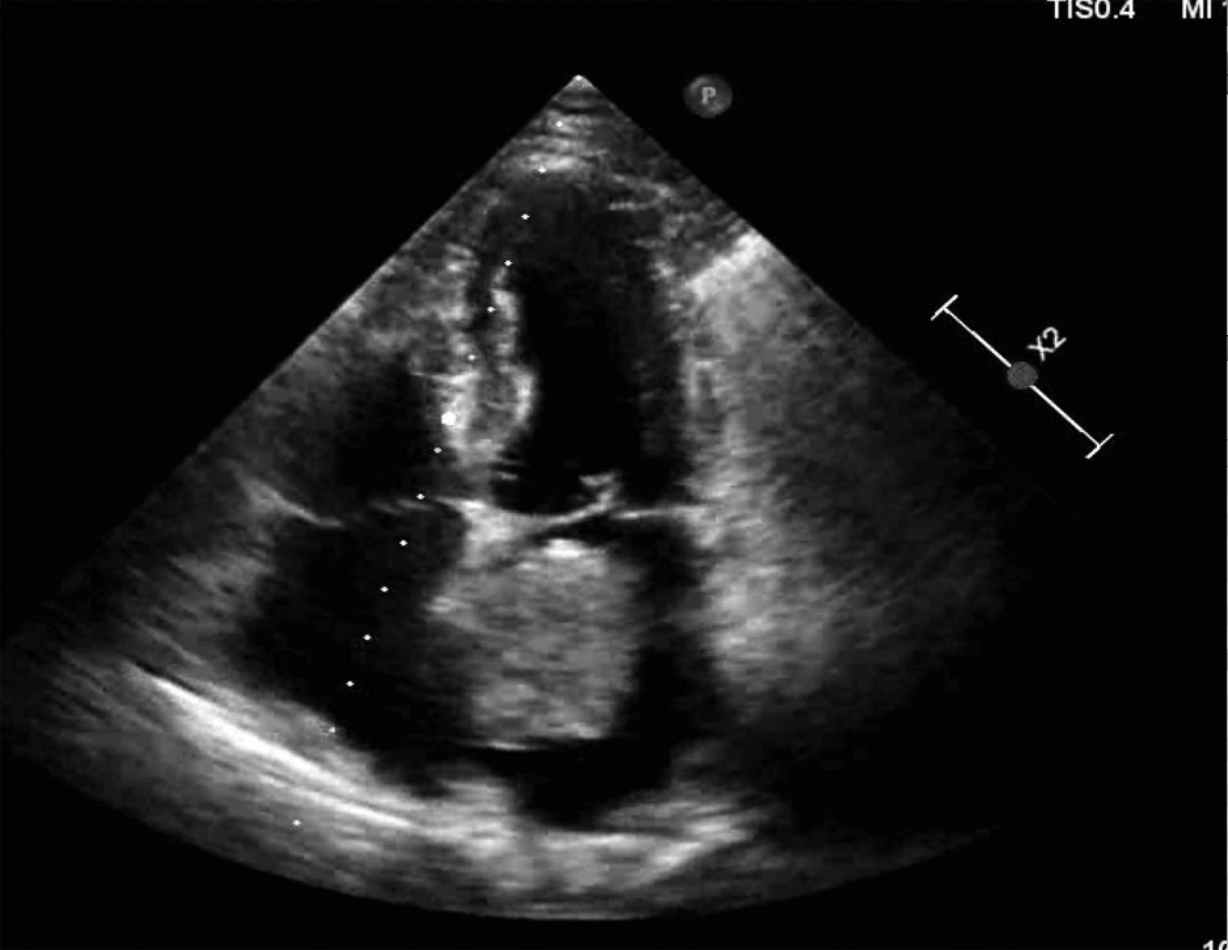
- Echocardiography demonstration 48 mm left atrial mass attached to the fossa ovalis of the /atrial septum typical for cardiac myxoma.

Meningitis and encephalitis test resulted in a negative cerebrospinal fluid sample, and a computed tomography (CT) brain scan revealed a sellar/suprasellar mass with mild ventricular dilation. Pituitary macroadenoma with apoplexy was confirmed by magnetic resonance imaging along with old subarachnoid and intraventricular hemorrhages (Figure 1). The results of laboratory investigations are shown in Table 2. A left atrial mass, a lung mass, bilateral hilar and mediastinal lymphadenopathy, a large thyroid lesion, a small left adrenal mass, and multiple lytic bone lesions were discovered on CT scans of the chest and abdomen (Figures 2). Echocardiography confirmed the left atrial mass attachment to the fossa ovalis, confirming the diagnosis of atrial myxoma (Figure 3). Malignancy screening, including stool occult blood, tumor markers, and protein electrophoresis, was negative during the patient’s hospital stay. Furthermore, fine-needle aspiration of thyroid lesions revealed follicular cells without malignancy. Due to the patient’s rapid clinical deterioration, neither a lung lesion biopsy nor a myxoma resection was possible.

**Table 2:** - Patient’s biochemical profile.

Laboratory investigations	Results
Serum sodium	135 (136-145 Nmol/L)
Serum potassium	4.5 (3.6-5.2 mmol/L)
Serum chloride	103 (96-106 mmol/L)
TSH	0.101 (0.45-4.5 mU/L)
Free T4	8.33 (12-30 pmol/L)
Free T3	2.57 (2-7 pmol/L)
Random glucose	3.9 (3.9-5.6 mmol/L)
Random cortisol	2070 (136-636 Nmol/L)
ACTH	1.11 (1.6-13.9 pmol/L)
GH	0.093 (0.4-10 Ng/ml)
IGF	15 (85-95 Ng/ml)
FSH	8.93 (1.5-12.4 IU/L)

TSH: thyroid stimulating hormone, T4: thyroxine, T3: triiodothyronine, ACTH: adrenocorticotropic hormone, GH: growth hormone, IGF: insulin-like growth factor, FSH: follicular stimulating hormone

### Therapeutic assessment

Antibacterial and antiviral treatments were started then discontinued after meningitis and encephalitis results came back negative. Intravenous hydrocortisone and oral levothyroxine were started. An intraventricular drain was inserted. Hemodynamic stabilization and invasive ventilation, as well as rate control medication, were used to treat newly diagnosed atrial fibrillation.

### Follow-up and outcome

On day 65 of hospitalization, the patient’s level of consciousness deteriorated, which was attributed to the hemorrhagic transformation of right occipital acute ischemic infarction. Death was announced 9 days later.

## Discussion

Although neurological symptoms are common in cardiac myxoma, the combination of subarachnoid and intraventricular haemorrhages, ischemic stroke, pituitary adenoma, and pituitary apoplexy is extremely uncommon.^7,8^ Despite the lack of histopathological confirmation, the presence of cardiac myxoma with non-secreting pituitary macroadenoma, multiple bilateral thyroid nodules with follicular pathology, and left adrenal mass in our patient is highly suggestive of CNC.^9^ Carney complex is a rare autosomal dominant syndrome marked by skin tumors, pigmented lesions, myxomas, and a variety of endocrine tumors.^6^ Cardiac myxoma and non-functioning pituitary macroadenoma presenting initially with pituitary apoplexy has been rarely reported. Systemic embolizations can explain repeated neurological events over a short period of time which led to the patient’s deterioration and death. Associated lung and bone lesions have also been reported previously.^6,10^ The risk of embolization was linked to the tumor’s large size and irregular borders.^7^ In patients with multisystem involvement, secondary metastasis is frequently considered, but atrial myxoma with systemic embolization is rarely considered.

In conclusion, cardiac myxoma with systemic embolization is an excellent mimic for a wide range of infectious, inflammatory, and malignant diseases. When cardiac myxoma is associated with endocrine tumors and skin pigmentation, the rare CNC must be considered. The only curative treatment for cardiac myxoma and its systemic embolization is myxoma resection.
